# Clinical features, disease burden and impact on quality of life in participants with mitochondrial encephalomyopathy

**DOI:** 10.3389/fneur.2025.1585906

**Published:** 2025-07-18

**Authors:** John Sieh Dumbuya, Chuan Tian, Lin Deng, Bashir Ahmad, Xiuling Chen, Jun Lu

**Affiliations:** ^1^Department of Paediatrics, Affiliated Hospital of Guangdong Medical University, Zhanjiang, China; ^2^Department of Paediatrics, The 958 Hospital of the People’s Liberation Army, Chongqing, China; ^3^Department of Paediatrics, Haikou Affiliated Hospital of Central South University, Xiangya School of Medicine, Haikou, China

**Keywords:** mitochondrial encephalomyopathy, rare diseases, caregiver burden, financial burden, quality of life, assessment scales

## Abstract

**Background:**

Mitochondrial encephalomyopathy (ME) is a rare genetic disorder that significantly impacts participants’ quality of life and places emotional and financial burdens on caregivers. However, the dynamics between perceived financial burden, disability, and caregiver strain are not fully understood. This study aimed to explore the differences in perceived financial burden, QoL, disability levels, and caregiver burden among participants with ME.

**Methods:**

Between January and December 2023, we conducted a cross-sectional analysis of ME participants and their caregivers at Haikou Affiliated Hospital of Xiangya Medical College, Central South University. Multiple assessment scales, including CHU-9D, PedsQL, PHQ-9, and CBI, were used to evaluate disease burden, QoL, disability, and caregiver burden. Descriptive statistics and correlation coefficients were employed to assess the relationships between these factors.

**Results:**

A total of 27 participants with ME were identified, with a mean age of 10.14 years, 88.9% of whom were children. The cohort comprised 18 (66.7%) males and 9 (33.3%) females; MELAS and Leigh syndrome were the most common subtypes. Significant correlations were found between QoL scores and caregiver burden, with CHU-9D showing negative correlations with PHQ-9 and CBI and positive correlations with PedsQL and health utility scores. Additionally, 44.4% of participants reported severe financial burdens, and 57.7% of caregivers experienced moderate to severe levels of burden.

**Conclusion:**

Our findings highlight the complex relationships between financial strain, QoL, and caregiver burden in ME. This underscores the need for comprehensive, patient-centered care and targeted policy interventions to alleviate patient and caregiver burdens. Further research is essential to develop effective support systems and improve overall outcomes.

## 1 Introduction

Mitochondrial encephalomyopathy (ME) represents a group of inherited disorders arising from mitochondrial or nuclear DNA mutations, resulting in dysfunctions within the mitochondrial respiratory chain. These mutations hinder energy production, particularly in high-energy organs such as the brain, muscles, and heart. Consequently, individuals with ME may experience a range of clinical symptoms, including muscle weakness, seizures, stroke-like episodes, and progressive neurological decline (1, 2). The most common subtypes of ME include MELAS (Mitochondrial Encephalomyopathy, Lactic Acidosis, and Stroke-like Episodes), MERRF (Myoclonic Epilepsy with Ragged Red Fibers), and Leigh Syndrome. Each subtype is associated with distinct genetic causes and clinical manifestations (3–5). Despite significant advancements in genetic testing, the clinical presentation of ME remains highly variable, even among individuals with the same genetic mutation. This variability poses challenges for early diagnosis and timely intervention, which are critical for mitigating disease severity and enhancing patient outcomes (3, 6). Currently, management strategies emphasize symptom relief through medications and physical therapy (7–9). While current management strategies primarily focus on alleviating symptoms through medications, physical therapy, and supportive care, the disease’s profound impact on participants’ quality of life (QoL) and the burden on their caregivers has received less attention.

Mitochondrial encephalomyopathy profoundly affects participants’ QoL, limiting their ability to perform daily activities due to fatigue, pain, and mobility challenges, while cognitive impairments and neurological decline heighten these difficulties (10). Social isolation and psychological distress, including anxiety and depression, are common due to the illness’s progressive nature (11). Caregivers, too, endure substantial emotional and financial strain, often leading to burnout that affects family dynamics and diminishes overall QoL (12, 13). Recent studies have emphasized the importance of understanding the socioeconomic and psychosocial impact of rare diseases like ME, with a focus on financial strain, caregiving stress, and the overall well-being of affected individuals (14, 15). However, there is limited research examining the complex interplay between disease severity, financial burden, caregiver strain, and QoL in ME participants.

This study aims to investigate the relationships between disease burden, QoL, perceived financial strain, and caregiver burden in participants with ME and their caregivers. By examining these factors, we aim to underscore the need for improved diagnostics and support systems, with results that inform policy and clinical practices to better serve participants and their families.

## 2 Methods

### 2.1 Study design and population

This cross-sectional study focused on pediatric and adult participants diagnosed with mitochondrial encephalomyopathy (ME) as well as their caregivers. Participants were recruited from the Haikou Affiliated Hospital of Xiangya Medical College, Central South University, between January and December 2023. Eligibility was determined based on a confirmed genetic diagnosis of ME, which required specific clinical symptoms affecting the nervous and muscular systems, substantiated by genetic testing. This testing was complemented by clinical evaluations and MRI scans by experienced neurologists and MRI diagnostic specialists utilizing the Morava scale to assess the severity of the disease (16). The study population was further categorized by genetic subtype to investigate correlations between specific mutations, disease severity, quality of life, and caregiver burden. Participants diagnosed with ME forms outside the targeted subtypes or those who opted out of participation were excluded from the research. Only those participants who met the inclusion criteria and completed the survey were included in the final analysis.

### 2.2 Assessment procedures and respondent selection

Assessment tools and respondent selection were customized based on participants’ age and cognitive capacity. For those aged 6–18 years, the CHU-9D and PedsQL questionnaires were administered with self-reporting by participants when cognitively able. For children under 6, proxy reports from primary caregivers were used to assess quality of life dimensions, as young children typically cannot complete these instruments independently. Adult participants (≥18 years) completed assessments like the EQ-5D-3L, PHQ-9, and CBI themselves, provided their cognitive and neurological status allowed.

In cases of cognitive impairments or neurological deficits that hindered accurate self-reporting, proxies, mainly caregivers or family members familiar with the participant’s condition, provided responses. This ensured that the data accurately reflected participants’ health status or, when suitable, the caregiver’s observations. Proxy responses were gathered following standardized protocols, and clinicians reviewed them to ensure consistency and validity. This strategy aimed to enhance data completeness and accuracy across the diverse cognitive and age ranges of participants while acknowledging the limitations of proxy reporting in capturing individual emotional and psychosocial experiences.

### 2.3 Ethics statement

This cross-sectional study was conducted in accordance with the Declaration of Helsinki.

Ethical approval was obtained from the Institutional Review Board (IRB) of Haikou Affiliated Hospital. All participants or their caregivers provided written informed consent. The study adhered to strict ethical guidelines, ensuring the confidentiality and security of participants’ data.

### 2.4 Quality of life (QoL) outcome measures

#### 2.4.1 EQ-5D-3L

The EQ-5D-3L is a widely used tool for assessing health-related quality of life (HRQoL). It evaluates five essential health dimensions: Mobility, Self-Care, Usual Activities, Pain/Discomfort, and Anxiety/Depression. Each of these dimensions is categorized into three levels of severity: No problems, Some problems, and Extreme problems (17). The EQ-5D-3L produces a single index score by combining the responses across the five dimensions. This index score is derived from country-specific value sets that reflect the preferences and health perceptions of the population in a particular country. Utility values derived from EQ-5D-3L scores are then converted into standardized utility measures, referenced against national norms to allow comparisons across populations (18, 19). The resulting score ranges from less than 0 (indicating a health state worse than death) to 1 (representing perfect health). For children under the age of six, the questionnaire is completed by parents or guardians, acting as proxies to accurately represent the child’s health-related quality of life. This approach helps ensure the collection of reliable data when the child is too young to self-report.

#### 2.4.2 PedsQL

The Pediatric Quality of Life Inventory (PedsQL) is a widely recognized tool designed to assess health-related quality of life (HRQoL) in children and adolescents across various age groups. The PedsQL consists of 23 items that can be completed either by the child (self-report) or by a caregiver (proxy report), depending on the child’s age and ability to respond. The items are grouped into four core domains, which represent critical aspects of a child’s health and well-being: Physical Functioning (8 items), Emotional Functioning (5 items), Social Functioning (5 items) and School Functioning (5 items) (20). The responses to each item are rated using a 5-point Likert scale, ranging from 0 (never a problem) to 4 (almost always a problem). After the responses are collected, the scores are reverse-coded to ensure that higher scores reflect better quality of life. The individual scores for each domain are then transformed into a 0–100 scale, where a higher score corresponds to better HRQoL. The PedsQL scores were compared against national norms, which provide a reference point for understanding how a child’s HRQoL compares to the general population (21, 22).

#### 2.4.3 PHQ-9

The Patient Health Questionnaire-9 (PHQ-9) is a widely used, self-reported instrument designed to evaluate the severity of depressive symptoms in individuals. It is often employed in both clinical and research settings as a straightforward tool for identifying depression and assessing its severity over a defined period (23). The PHQ-9 consists of nine items that assess various depressive symptoms the individual has experienced over the past 2 weeks. Each item is rated on a 4-point Likert scale based on the frequency of symptoms during this period: 0 = Not at all; 1 = Several days; 2 = More than half the days; 3 = Nearly every day. The total score is calculated by summing the individual responses, yielding a score between 0 and 27. Higher scores indicate greater severity of depressive symptoms. The PHQ-9 score is further categorized into the following severity levels: 0–4: Minimal or none – Indicates little to no symptoms of depression; 5–9: Mild – Suggests mild depressive symptoms that may be manageable or transient; 10–14: Moderate – Indicates moderate symptoms, potentially warranting some form of intervention; 15–19: Moderately severe – Suggests more significant depressive symptoms, requiring clinical evaluation and possibly treatment, and 20–27: Severe depression – Indicates severe depression, typically requiring immediate clinical intervention and more intensive treatment. Given its widespread use, the PHQ-9 has been adapted for use in various languages and cultures, demonstrating its cross-cultural validity (24, 25).

#### 2.4.4 CHU-9D

The Child Health Utility 9-Dimensional (CHU-9D) tool is a specialized instrument developed to assess health-related quality of life (HRQoL) in children and adolescents, specifically those aged between 7 and 17. It provides a comprehensive measure of a child’s well-being by evaluating nine key dimensions of physical and emotional health (26, 27). CHU-9D evaluates the following nine dimensions of a child’s health, each of which represents an essential aspect of daily functioning: Worried, Sad, Pain, Tired, Annoyed, Schoolwork/Homework, Sleep, Daily Routine and Ability to Join in Activities, rated in 5-point scale (1: No problems; 2: Slight problems; 3: Moderate problems; 4: Severe problems; and 5: Very severe problems). The overall utility score is derived from a weighted combination of the individual scores, which results in a final utility score ranging from 0 to 1. A score of 0 represents the worst conceivable health state (equivalent to death), while a score of 1 represents perfect health. The derived CHU-9D utility values were then compared against national norms to offer context and enable comparisons across different populations (28). Proxy-reported questionnaires were used for children under the age of 7, who are often unable to complete the questionnaire themselves, completed by parents or guardians. This approach helps ensure that HRQoL is accurately assessed, even in younger children who may not be able to reliably self-report their experiences.

#### 2.4.5 CBI

The Caregiver Burden Inventory (CBI) is a comprehensive tool utilized to assess the burden experienced by caregivers of individuals with chronic conditions, including those with physical, mental, or cognitive impairments. The CBI evaluates caregiver burden across five distinct dimensions, each reflecting a different aspect of the caregiving experience: Time-Dependence, Developmental, Physical, Social, and Emotional burden (29). It consists of 24 items designed to capture various aspects of caregiver burden. Each item is rated on a 5-point Likert scale, where 0 = Not at all descriptive; 1 = Slightly descriptive; 2 = Moderately descriptive; 3 = Very descriptive; and 4 = Extremely descriptive. The caregiver’s responses across the 24 items are then summed up to generate a total score for each dimension, with scores ranging from 0 to 96. A higher score in any dimension indicates a greater degree of burden in that particular area. The total score is then used to categorize the overall caregiver burden into different levels, which helps assess the intensity of the burden experienced: 0–20: Little or no burden; 21–40: Mild to moderate burden; 41–60: Moderate to severe burden; and 61–96: Severe burden.

#### 2.4.6 OSSS-3

The Oslo Social Support Scale (OSSS-3) is a concise, three-item self-report measure designed to assess an individual’s perceived social support. Social support significantly influences mental and physical health, and the OSSS-3 offers a quick and effective method for measuring this essential aspect of well-being. The OSSS-3 examines three key dimensions of social support:

1. Number of Close Confidants – This item gauges the individual’s perception of how many people they can depend on for emotional support, such as friends or family members with whom they can confide.
2. Interest and Concern from Others – This item assesses how much the individual feels cared for and supported by others, including the degree of emotional investment and attention displayed by their social network.
3. Practical Help from Neighbors – This item evaluates the extent to which the individual receives practical assistance from neighbors or the local community, such as help with tasks or emergencies (30).

The OSSS-3 employs a straightforward scoring system to measure the perceived level of social support. Each item is rated on a five-point Likert scale: 1 = Very little; 2 = Little; 3 = Moderate; 4 = Much; and 5 = Very much. The individual’s responses to the three items are summed to yield a total score ranging from 3 to 14, with a higher score indicating a greater perceived level of social support. The total score is categorized into the following levels of social support: 3–8: Poor social support; 9–11: Moderate social support; and 12–14: Strong social support.

### 2.5 Participants classification for subgroup analysis

To enable more detailed statistical analyses, participants were grouped based on key characteristics: age (children vs. adults), clinical disease types (e.g., MELAS, Leigh syndrome), and genetic mutations (e.g., m.3243A > G, other mutations). This stratification sought to identify differences in quality of life, disease burden, and psychological measures across patient populations and disease subtypes, allowing for a deeper understanding of the heterogeneity within mitochondrial encephalomyopathy.

### 2.6 Statistical analysis

All participants who satisfied the inclusion criteria were incorporated into the analysis. Categorical variables were reported as counts and percentages, whereas continuous variables were presented as means and standard deviations. Spearman’s correlation coefficient was utilized to evaluate the relationships between continuous variables. The ANOVA test was employed to compare QoL measurements across varying levels of perceived financial burden and disability. All statistical analyses were conducted using IBM SPSS Statistics for Windows, Version 22 (IBM Corp., Armonk, NY, United States), with a significance level set at *p* < 0.05.

## 3 Results

### 3.1 General characteristics of socio-demographics

Table 1 displays key characteristics of 27 participants diagnosed with distinct mitochondrial encephalomyopathy (ME). The average age was 10.14 years (SD 9.24), with the youngest patient being just 1 month old and the oldest being 36 years old. There were 18 male participants (66.7%) and nine female participants (33.3%), with the majority being children (88.9%). The most identified ME subtypes were MELAS and Leigh syndrome (6 participants each, 22.2%), followed by COXPD1 (5 participants, 18.5%). Additionally, there were two participants each for MERRF, EMPF1, and COQ10D (7.4% each). In terms of age range, 63.0% of the participants (17/27) were 0–9 years old, 25.9% (7/27) were 10–19 years old, 7.4% (2/27) were 20–29 years old, and 3.7% (1/27) were 30–39 years old. Approximately 25.9% of the participants had a family history of ME. Additional details on the number of referrals and inpatient visits are available in Table 1.

**Table 1:** Participants characteristics.

Variable	*n* (%) or Mean ± SD
Sex	
Male	18 (66.7)
Female	9 (33.3)
Age (years)	10.14 ± 9.24
Age range	
0–9 years	17 (63.0)
10–19 years	7 (25.9)
20–29 years	2 (7.4)
30–39 years	1 (3.7)
ME subtypes	
adPEO	1 (3.7)
COQ10D	2 (7.4)
COXPD	5 (18.5)
ECHSID	1 (3.7)
EMPF1	2 (7.4)
Leigh syndrome	6 (22.2)
LHON	1 (3.7)
MCIDN20	1 (3.7)
MELAS	6 (22.2)
MERRF	2 (7.4)
Diagnostic odyssey	
<1 yr	15 (55.6)
1–3 yrs	6 (22.2)
>3 yrs	6 (22.2)
Diagnostic delay (yrs)	1.91 ± 2.64
Family history	
No	20 (74.1)
Yes	7 (25.9)
Number of referrals	
0 times	14 (519)
1–3 times	12 (44.4)
4–7 times	1 (3.7)
Number of inpatient visits	
1–5 times	19 (70.4)
6–10 times	4 (14.8)
11–15 times	4 (14.8)
Rehabilitation	
No	15 (55.6)
Yes	12 (44.4)
Debt incurred	
No	3 (11.1)
Yes	24 (88.9)
Amount of debt incurred each year (CNY)	
0–30,000	7 (25.9)
31,000–60,000	9 (33.3)
61,000–100,00	4 (14.8)
101,000–200,000	7 (25.9)
Hospitalization (CNY)	77,137.76 ± 105,250.76

ME, mitochondrial encephalomyopathy; adPEO, autosomal dominant progressive external ophthalmoplegia; COQ10D, primary coenzyme Q10 deficiency; COXPD, combined oxidative phosphorylation deficiency; ECHSID, mitochondrial short-chain enoyl-coA hydratase 1 deficiency; EMPF1, encephalopathy due to defective mitochondrial and peroxisomal fission 1; LHON, Leber’s hereditary optic neuropathy; MCIDN2O, mitochondrial complex 1 deficiency, nuclear type 20; MELAS, mitochondrial encephalopathy, lactic acidosis, and stroke-like episodes; MERRF, myoclonic epilepsy with ragged red fibers; SD, standard deviation; CNY, Chinese yuan.

**Figure 1:**
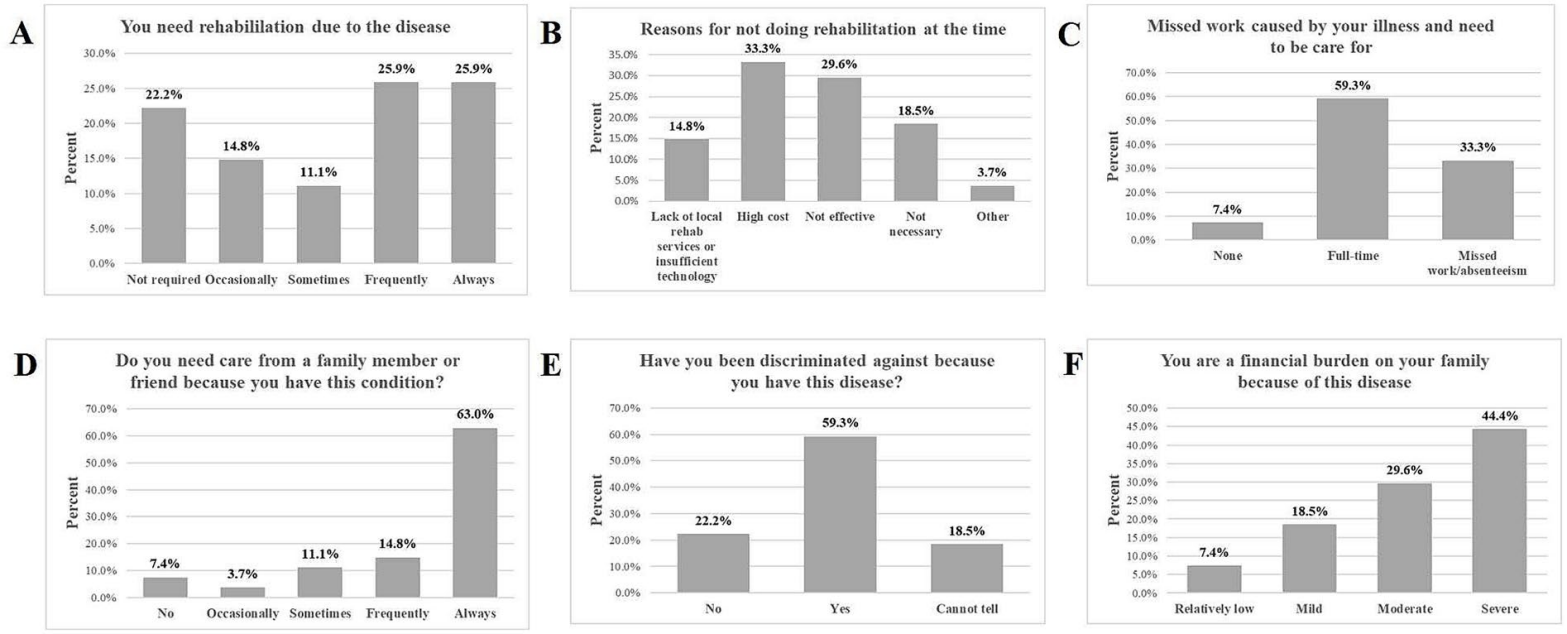
The impact of ME on rehabilitation, work, discrimination, and financial burden. **(A)** Illustrates the proportion of participants who reported their need for rehabilitation due to the disease. **(B)** Highlights the reasons why some participants did not engage in rehabilitation. **(C)** Displays the extent of missed work due to the illness and the need for care. Panel **(D)** shows how many participants required care from family or friends. **(E)** Illustrates the extent to which participants have experienced discrimination because of their illness. **(F)** Presents how participants perceive their financial burden on their families.

Among the 27 participants, 12 (44.4%) opted for rehabilitation intervention. Of those seeking rehabilitation due to their medical condition, 25.9% required it always and frequently, 11.1% required it sometimes, and 14.8% required it occasionally. For individuals who did not pursue rehabilitation programs, 33.3% cited high costs, 29.6% found it ineffective, and 14.8% lacked local rehabilitation services or advanced technology (Figures 1A,B). A significant 59.3% of participants required full-time care from their family members, while 33.3% of participants reported missing work or being absent to care for family members (Figure 1C). Among those who needed care from a family member or friend, 62.9% needed it always, 14.8% needed it frequently, 11.1% needed it sometimes, 3.7% required it occasionally, and 7.4% did not need it (Figure 1D). In our study, 16 (59.3%) of respondents, mainly caregivers of pediatric participants or adult participants themselves, reported discrimination related to their or their family members’ condition. In comparison, 5 (18.5%) could not tell, and 6 (22.2%) did not report any discrimination (Figure 1E).

### 3.2 Disease burden and financial impact

The average hospitalization cost was 77,137.76 CNY, with an average insurance coverage of 31,828.09 CNY, representing 41.3% of the total cost. As indicated in Table 1, 24 (88.9%) reported incurring debt due to medical expenses, with some independent adult participants also contributing to this figure, with 33.3% having 30,000–60,000 CNY debt, 25.9% having 0–30,000 CNY, 25.9% having 100,000–200,000 CNY, and 14.8% having 60,000–100,000 CNY debt. The average diagnostic delay was 1.9 (SD 2.6) years, ranging from 0 to 10.8 years. The diagnostic delay was defined as the time from the first symptom onset to the final diagnosis of ME. The diagnostic odyssey took an average of 1.67 (SD 0.83) years to reach a definitive diagnosis. More than half (55.6%) of the participants were diagnosed within a year, 22.2% were diagnosed within 1 to 3 years, and the remaining 22.2% took longer than 3 years to be diagnosed (Table 1).

**Figure 2:**
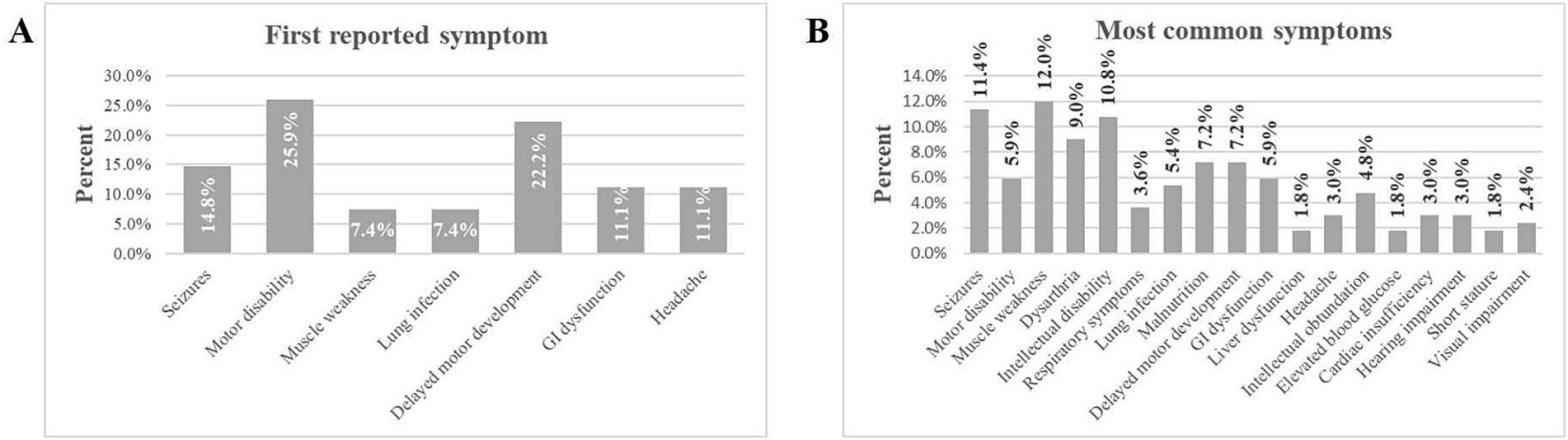
Percentage of symptoms experienced by individuals with ME. **(A)** Displays the first reported symptom experienced by participants. **(B)** Lists the most common symptoms experienced by participants.

On average, participants reported 3.48 (SD 2.06) distinct symptoms at the onset of the disease. The most common first reported symptom was motor disability (25.9%), followed by delayed motor development (22.2%). Other frequently reported symptoms included seizures (14.8%), gastrointestinal dysfunction (11.1%) and headache (11.1%). Symptoms like lung infection (7.4%) and muscle weakness (7.4%) were less commonly the first reported (Figure 2A). Thus, muscle-related issues (delayed motor development) dominate as the first signs of disease, suggesting they may serve as early indicators of this condition. Subsequently, seizures (11.4%) and muscle weakness (12.0%) were the most reported symptoms overall. Other prevalent symptoms included intellectual disability (10.8%); dysarthria (speech difficulties, 9.0%); malnutrition (7.2%), and delayed motor development (7.2%). Symptoms such as motor disability (5.9%), gastrointestinal (GI) dysfunction (5.9%), and lung infection (5.4%) were moderately common. Less common symptoms included headache, respiratory symptoms, hearing impairment, and cardiac insufficiency (3.0% or less) (Figure 2B). Thus, neuromuscular symptoms (e.g., motor disability, seizures, muscle weakness) were the most frequently reported overall, emphasizing the need to monitor these signs for early diagnosis and management.

### 3.3 Phenotype and genotype analysis

**Figure 3:**
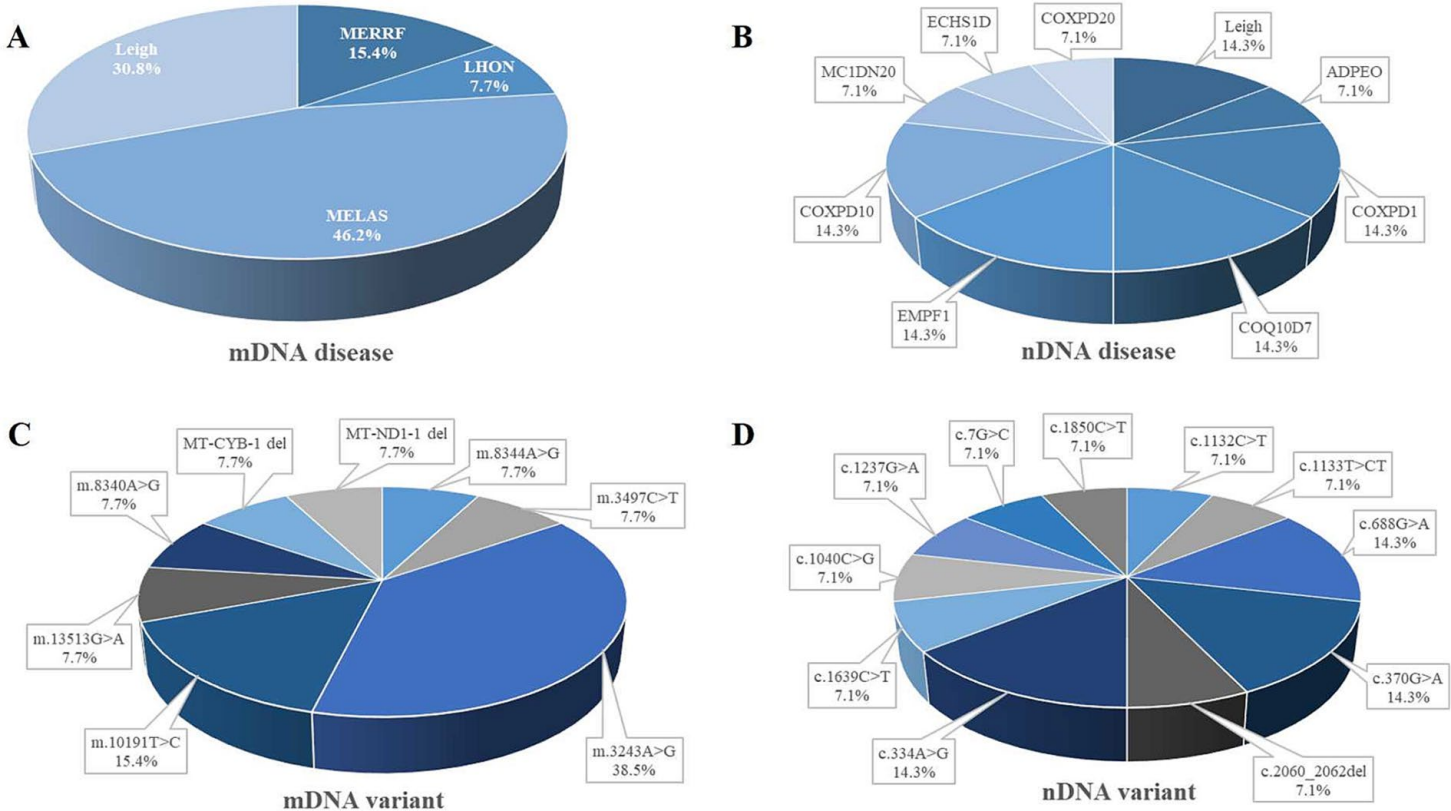
Distribution of ME subtypes and variant. Panel **(A)** shows the distribution of different mitochondrial DNA (mtDNA) diseases. **(B)** Represents the distribution of nuclear DNA (nDNA) diseases. Panel **(C)** shows the distribution of mitochondrial DNA variants. **(D)** Represents the distribution of nuclear DNA variants. adPEO, autosomal dominant progressive external ophthalmoplegia; COQ10D, primary coenzyme Q10 deficiency; COXPD, combined oxidative phosphorylation deficiency; ECHSID, mitochondrial short-chain enoyl-coA hydratase 1 deficiency; EMPF1, encephalopathy due to defective mitochondrial and peroxisomal fission 1; LHON, Leber’s hereditary optic neuropathy; MCIDN2O, mitochondrial complex 1 deficiency, nuclear type 20; MELAS, mitochondrial encephalopathy, lactic acidosis, and stroke-like episodes; ME, mitochondrial encephalomyopathy; MERRF, myoclonic epilepsy with ragged red fibers.

Mitochondrial encephalomyopathy with lactic acidosis and stroke-like episodes (MELAS) was identified as the most prevalent mitochondrial DNA (mtDNA) disease, accounting for 46.2% of cases within the cohort, Leigh syndrome accounted for 30.8% of individuals; myoclonic epilepsy with ragged red fibers (MERRF) represented 15.4% of participants. Leber’s hereditary optic neuropathy (LHON) was 7.7% of the cases (Figure 3A). Among nuclear DNA (nDNA)-related diseases, combined oxidative phosphorylation deficiency (COXPD1), encephalopathy due to defective mitochondrial and peroxisomal fission 1 (EMPF1), and primary coenzyme Q10 deficiency (COQ10D7) were the most frequently encountered, each constituting 14.3% of cases, and were linked to metabolic or mitochondrial dysfunctions. Leigh syndrome’s prominence in nDNA-related diseases highlighted its unique dual classification across both DNA types (Figure 3B). In the analysis of mtDNA variants, the m.3243A > G mutation was the most prevalent, evident in 38.5% of cases associated with MELAS and the m.10191 T > C variant was 15.4%. Several additional variants accounted for 7.7% each (Figure 3C). Concerning nDNA variants, three mutations—c.334A > G, c.370G > A, and c.688G > A—were the most common, each representing 14.3% of cases. Additionally, other variants contributed 7.1% each (Figure 3D). mtDNA mutations primarily included point mutations or deletions within mitochondrial genes, whereas nDNA variants presented as point mutations, deletions, or compound variants in nuclear genes (Table 2).

**Table 2:** Genetic variants and clinical features.

ID	Sex	Disease	ME type	Variant and location	First reported symptom	Main symptoms	Prognosis/Outcome
P1	Male	MERRF	mNDA	MT-TK m.8344A > G	Motor retardation	Seizures, motor retardation, weakness, dysarthria, mental retardation, recurrent pneumonia, malnutrition	Stable
P2	Male	LS	nDNA	PDHA1 c.1132C > T	Motor retardation, facial weakness	Motor retardation, seizures, limb weakness, lung infections	Death
P3	Male	ADPEO	nDNA	C10orf2 c.1133 T > CT	Muscle weakness	muscle weakness, respiratory symptoms, malnutrition	Exacerbated
P4	Male	COXPD1	nDNA	GFM1 c.688G > A	Delayed motor development	Seizures, delayed motor development, abnormal liver function, malnutrition, gastrointestinal dysfunction, lung infections	Improved
P5	Male	LHON	mNDA	MT-ND1 m.3497C > T	Motor retardation, facial weakness	Motor retardation, headache, muscle weakness, muscle atrophy, mental obtundation	Death
P6	Female	MELAS	mNDA	MT-TL1 m.3243A > G	Limb weakness, delayed motor development	Stroke-like episodes, delayed motor development, limb weakness, hypotonia, amyotrophy, malnutrition, dysarthria, mental retardation, gastrointestinal disorders, lung infection, liver dysfunction, cardiac insufficiency	Exacerbated
P7	Male	COQ10D7	nDNA	COQ4 c.370G > A	Seizures	seizures, delayed motor development, dysarthria, mental retardation, hearing impairment, malnutrition, recurrent lung infections, increased muscle tone, gastrointestinal dysfunction	Exacerbated
P8	Female	COQ10D7	nDNA	COQ4 c.370G > A	Seizures, delayed motor development	Seizures, delayed motor development, dysarthria, mental retardation	Stable
P9	Female	LS	mNDA	MT-ND3 m.10191 T > C	Motor retardation, facial weakness	Motor retardation, facial weakness, short stature, seizures, mental retardation	Death
P10	Male	EMPF1	nDNA	DNM1L c.2060_2062delTAG	Motor retardation	seizures, motor retardation, dysarthria, mental retardation, muscle weakness	Exacerbated
P11	Male	COXPD10	nDNA	MTO1 c.334A > G	Lung infections	Delayed motor development, mental retardation, lung infection, gastrointestinal dysfunction, elevated blood glucose, muscle weakness, cardiac insufficiency	Death
P12	Female	COXPD10	nDNA	MTO1 c.334A > G	Delayed motor development, limb weakness	Delayed motor development, dysarthria, mental retardation, limb weakness, malnutrition	Improved
P13	Male	LS	mNDA	MT-ND1-1 del	Seizures	Seizures, dysarthria, motor retardation, lung infections	Improved
P14	Male	LS	nDNA	PC c.1639C > T	Gastrointestinal dysfunction	Dysarthria, delayed motor development, mental retardation, limb weakness, feeding difficulties	Improved
P15	Male	LS	mNDA	MT-ND5 m.13513G > A	Delayed motor development, muscle weakness	Dysarthria, delayed motor development, mental retardation, limb weakness, malnutrition, seizures, lung infections	Death
P16	Female	EMPF1	nDNA	DNM1L c.1040C > G	Motor retardation, limb weakness	Seizures, motor retardation, limb weakness, dysarthria, mental retardation, short stature, gastrointestinal dysfunction	Death
P17	Female	MC1DN20	nDNA	ACAD9 c.1237G > A	Delayed motor development	Pulmonary infection, delayed motor development, cardiac insufficiency/hypertrophic cardiomyopathy	Death
P18	Male	ECHS1D	nDNA	ECHS1 c.7G > C	Delayed motor development	Delayed motor development, dysarthria, limb weakness, hearing impairment, malnutrition, visual impairment, mental retardation	Stable
P19	Female	COXPD1	nDNA	GFM1 c.688G > A	Gastrointestinal dysfunction, mental obtundation	Gastrointestinal dysfunction (feeding difficulties), mental obtundation, malnutrition, abnormal liver function, muscle weakness (limbs)	Death
P20	Male	MELAS	mNDA	MT-TL1 m.3243A > G	Headache, limb weakness	Limb weakness, headache, visual disturbances, stroke-like attacks, mental retardation, dysarthria, motor retardation, hypertrophic cardiomyopathy	Death
P21	Male	LS	mNDA	MT-ND3 m.10191 T > C	Headache, seizures	Seizures, headaches, motor retardation	Exacerbated
P22	Male	MELAS	mNDA	MT-TL1 m.3243A > G	Headaches, visual impairment, hearing impairment	Headache, visual impairment, hearing impairment, seizures, mental retardation, mild depression, gastrointestinal disorders	Improved
P23	Female	MELAS	mNDA	MT-TL1 m.3243A > G	Gastrointestinal dysfunction, headache	Seizures, malnutrition, mental retardation, headache, gastrointestinal dysfunction, hearing impairment, limb weakness, visual impairment, renal insufficiency	Stable
P24	Male	MERRF	mNDA	MT-TK m.8340A > G	Delayed motor development	Seizures, muscle weakness/hypotonia, delayed motor development, dysarthria, malnutrition, mental retardation, respiratory infections	Stable
P25	Male	COXPD20	nDNA	VARS2 c.1850C > T	infection/respiratory symptoms, mental obtundation	Respiratory symptoms/pulmonary infection, elevated blood glucose, seizures, hypotonia, cardiac insufficiency	Death
P26	Male	MELAS	mNDA	MT-TL1 m.3243A > G	Delayed motor development, dysarthria	Mental retardation, delayed motor development, dysarthria, short stature, malnutrition, limb weakness, seizures, gastrointestinal dysfunction, lung infection, hearing impairment, elevated blood glucose	Death
P27	Female	MELAS	mNDA	MT-CYB-1 del	Seizures	Pulmonary infection, seizures, dysarthria, motor retardation, limb weakness, mental obtundation	Not applicable

adPEO, autosomal dominant progressive external ophthalmoplegia; COQ10D, primary coenzyme Q10 deficiency; COXPD, combined oxidative phosphorylation deficiency; ECHSID, Mitochondrial short-chain enoyl-coA hydratase 1 deficiency; EMPF1, encephalopathy due to defective mitochondrial and peroxisomal fission 1; LHON, Leber’s hereditary optic neuropathy; LS, Leigh syndrome; MCIDN2O, Mitochondrial complex 1 deficiency, nuclear type 20; MELAS, Mitochondrial encephalopathy, lactic acidosis, and stroke-like episodes; ME, mitochondrial encephalomyopathy; MERRF, myoclonic epilepsy with ragged red fibers; mDNA, mitochondrial DNA; nDNA, nuclear DNA.

### 3.4 Quality of life scores

Most participants completed various questionnaires such as CHU-9D (*n* = 13, 48.1%), EQ-5D-3L (*n* = 12, 44.4%), OSSS-3 (*n* = 23, 85.2%), PHQ-9 (*n* = 14, 51.9%), and PedsQL (*n* = 13, 48.1%), while caregivers completed the CBI (*n* = 26, 96.3%). QoL scores were significantly lower than national norms across all scales. The mean score (SD) for CHU-9D was 8.92 (6.30), EQ-5D-3L 9.08 (2.43), OSSS-3: 6.43 (1.88), PHQ-9: 5.57 (6.01), CBI: 40.15 (16.73), and PedsQL total score was 55.03 (26.84) (Table 3).

**Table 3:** Quality of life assessment scales.

QoL scales	Mean ± SD	Ref*
QoL scales		
CHU-9D total score	8.92 ± 6.30	
Utility score	0.57 ± 0.30	0.88 ± 0.10
EQ-5D-3L total score	9.08 ± 2.43	
Utility score	0.55 ± 0.26	0.92 ± 0.13
PHQ-9 total score	5.57 ± 6.01	5.6 ± 4.95
OSSS-3 total score	6.43 ± 1.88	10.25 ± 2.40
CBI total score	**40.15 ± 16.73**	35.26 ± 12.30
Time-dependence burden	14.12 ± 4.56	
Developmental burden	14.46 ± 5.53	
Physical burden	9.38 ± 4.53	
Social burden	7.96 ± 5.66	
Emotional burden	1.46 ± 2.30	
PedsQL total score	**55.03 ± 26.84**	87.36 ± 7.23
Physical functioning	46.39 ± 32.52	
Emotional functioning	89.23 ± 17.78	
Social functioning	43.46 ± 42.64	
School functioning	50.58 ± 43.60	
Psychosocial functioning	60.90 ± 24.43	

Number of participants who completed each scale: CHU-9D (*N* = 13), EQ-5D-3L (*N* = 12), OSSS-3 (*N* = 23), PHQ-9 (*N* = 14), CBI (*N* = 26), PedsQL (*N* = 13). CHU-9D, Child health utility-9 dimension; EQ-5D-3L, European quality of life 5-dimension 3-level; OSSS-3, Oslo social support scale 3; QoL, Quality of life; CBI, Caregiver burden inventory; PedsQL, Pediatric quality of life inventory; SD, Standard deviation; VAS, Visual analog scale; *National norm values. Bold values refer to the total mean score of the overall scales, since both CBI and PedsQL are further divided into sub-scales or domains each.

**Figure 4:**
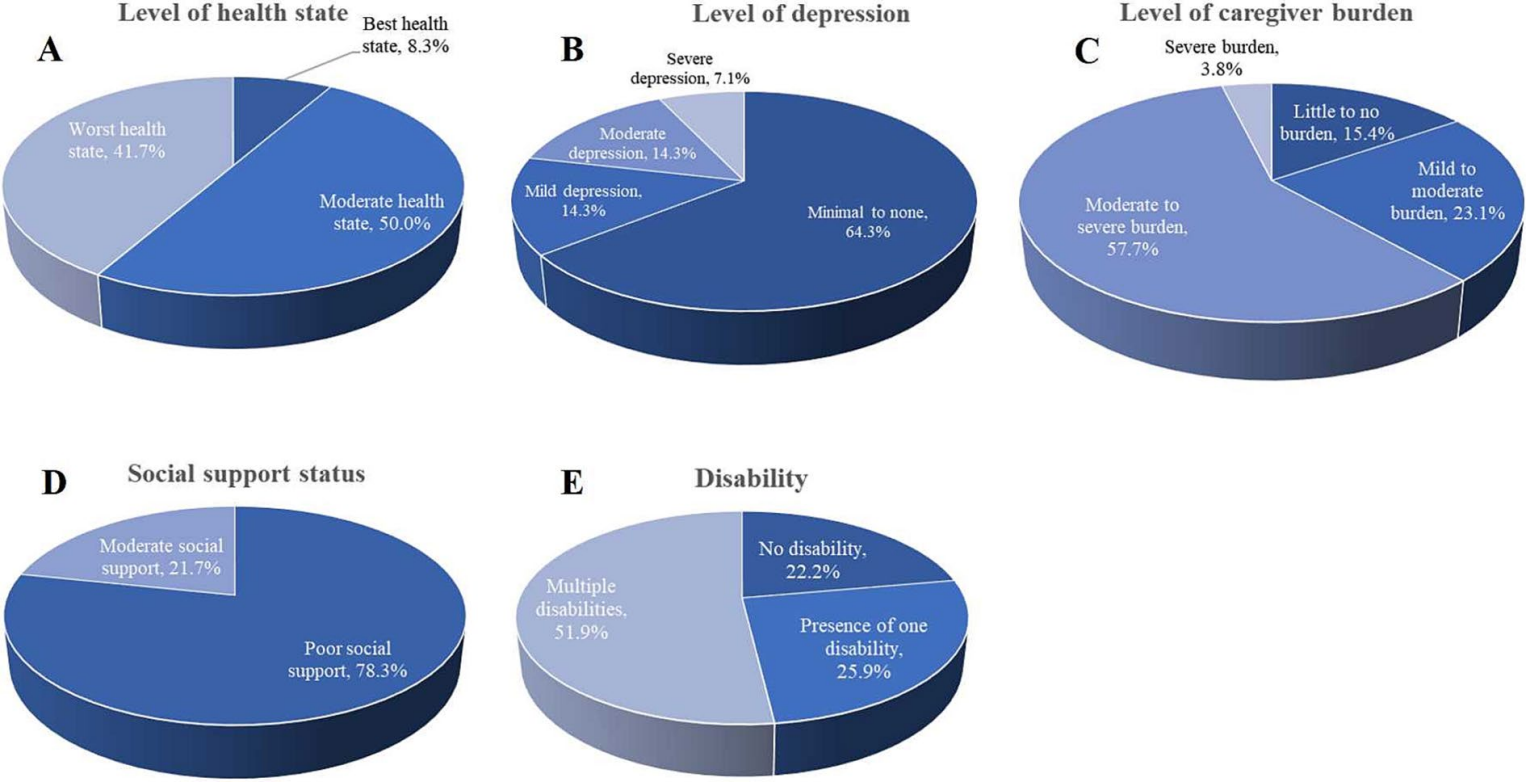
Level of health state, depression, caregiver burden, and social support status. **(A)** The level of health state was assessed using the EQ-5D-3L, showing that 41.7% of participants reported their health as being in the worst state. **(B)** Depression levels were assessed using the PHQ-9, most participants (64.3%) reported minimal to no depression, but 7.1% experienced severe depression. **(C)** The level of caregiver burden was assessed using the CBI. **(D)** Social support was measured using the OSSS-3 scale, and **(E)** presents the distribution of disabilities among the respondents.

The assessment of health status using the EQ-5D-3L revealed that 41.7% of respondents reported their health as being in the worst state, and 50.0% described their health as moderate. Only a small segment (8.3%) indicated they were in the best health state (Figure 4A). This finding suggests that most individuals are facing poor to moderate health, highlighting significant challenges within the population. The PHQ-9 score indicated that a significant portion of participants experienced mild to moderate depressive symptoms (64.3%), 14.3% experienced mild or moderate depression, and 7.1% reported severe depression (Figure 4B). Regarding caregiver burden, evaluated using the Caregiver Burden Inventory (CBI), 57.7% reported experiencing a moderate to severe burden, while 23.1% described their burden as mild to moderate. Only 15.4% felt a little to no burden, and 3.8% reported experiencing a severe burden (Figure 4C). Furthermore, a significant majority (78.3%) of respondents reported experiencing poor social support, as measured by the OSSS-3 scale. In contrast, 21.7% reported having moderate social support (Figure 4D). This suggests that social isolation or inadequate social support is a pressing concern that could exacerbate both health and caregiving challenges.

### 3.5 Correlation of QoL assessment scales and caregiver burden

Spearman’s correlation analysis revealed several significant relationships. The diagnostic delay showed a negative correlation with PHQ-9 total score (rho = −0.492, *p* < 0.037), CBI total score (rho = −0.370, *p* < 0.031), developmental burden (rho = −0.420, *p* < 0.016), physical burden (rho = −0.388, *p* < 0.032) and the level of caregiver burden (rho = −0.410, *p* < 0.019). Similarly, the number of symptoms correlated negatively with PHQ-9 total score (rho = −0.643, *p* < 0.007), CBI total score (rho = −0.482, *p* < 0.006), developmental burden (rho = −0.416, *p* < 0.017) and physical burden (rho = −0.501, *p* < 0.005). The CHU-9D demonstrated moderate negative correlations with the PHQ-9 total score (rho = −0.554, *p* < 0.031), strong negative correlation with the CBI total score (rho = −0.895, *p* < 0.001), and levels of caregiver burden (rho = −0.935, *p* < 0.000), time-dependence burden (rho = −0.682, *p* < 0.005), developmental burden (rho = −0.698, *p* < 0.004), physical burden (rho = −0.794, *p* < 0.001), and emotional burden (rho = −0.755, *p* < 0.001). Additionally, the CHU-9D strongly positively correlated with the PedsQL total score (rho = 0.702, *p* < 0.004) and its subdomains: physical functioning (rho = 0.524, *p* < 0.033), emotional functioning (rho = 0.565, *p* = 0.022), social functioning (rho = 0.666, *p* < 0.006), school functioning (rho = 0.746, *p* < 0.011), and psychosocial functioning (rho = 0.736, *p* < 0.002). It also showed a strong positive correlation with health utility score (rho = 0.857, *p* < 0.000) and levels of health state (rho = 0.742, *p* < 0.004). Furthermore, the OSSS-3 total score showed moderate positive correlations with the health utility score (rho = 0.512, *p* < 0.044) and PedsQL emotional functioning (rho = 0.564, *p* < 0.022). It also strongly negatively correlated with the level of health state (rho = −0.716, *p* < 0.004).

The PHQ-9 total score exhibited positive correlations with the CBI total score (rho = 0.690, *p* < 0.005), developmental burden (rho = 0.553, *p* < 0.025), physical burden (rho = 0.672, *p* < 0.006), social burden (rho = 0.510, *p* < 0.038), and level of caregiver burden (rho = 0.700, *p* < 0.004). It also showed moderate negative correlations with the PedsQL total score (rho = −0.522, *p* < 0.041), as well as with the subscales of emotional functioning (rho = −0.60, *p* < 0.007), social functioning (rho = −0.530, *p* < 0.038), and psychosocial functioning (rho = −0.575, *p* < 0.025). The CBI total score was negatively correlated with the PedsQL total score (rho = −0.702, *p* < 0.004) and its subdomains, which include physical function (rho = −0.542, *p* < 0.028), emotional functioning (rho = −0.599, *p* < 0.015), social functioning (rho = −0.665, *p* < 0.007), school functioning (rho = −0.726, *p* < 0.013), and psychosocial functioning (rho = −0.735, *p* < 0.002). It was positively correlated with the level of health state (rho = 0.695, *p* < 0.009) and the level of depression (rho = 0.517, *p* < 0.035). The PedsQL total score exhibited a positive correlation with health utility score (rho = 0.714, *p* < 0.007) and a negative correlation with developmental burden (rho = −0.806, *p* < 0.000), physical burden (rho = −0.699, *p* < 0.004), emotional burden (rho = −0.663, *p* < 0.007), and the level of caregiver burden (rho = −0.672, *p* < 0.006).

### 3.6 Correlation of participants’ perceived financial burden and their caregivers with QoL scales

Participants were surveyed to assess the impact of their condition on their family’s financial well-being. Results indicated that 44.4% (12/27) reported severe perceived financial burden, 29.6% (9/27) reported moderate financial burden, 18.5% (5/27) reported mild financial burden, and 7.4% (2/27) reported relatively low financial burden (Figure 1F).

The perceived financial burden was found to have a moderate negative correlation with the CHU-9D total score (rho = −0.658, *p* < 0.007), a moderate negative correlation with the utility health score (rho = −0.566, *p* < 0.027), and negatively correlated with diagnostic delay (rho = −0.399, *p* < 0.020). Moreover, the perceived financial burden showed a moderate negative correlation with the PedsQL total scores (rho = −0.555, *p* < 0.024), as well as with its subdomains: social functioning (rho = −0.504, *p* < 0.039), school functioning (rho = −0.619, *p* < 0.038), and psychosocial functioning (rho = −0.555, *p* < 0.024). On the other hand, the perceived financial burden was found to have a positive correlation with disability (rho = 0.339, *p* < 0.042). The PHQ-9 total score and the level of depression were moderately positively correlated (rho = 0.624, *p* < 0.009; rho = 0.461, *p* < 0.048, respectively), as was the CBI total score and the level of caregiver burden (rho = 0.526, *p* < 0.003, rho = 0.503, *p* < 0.004, respectively). Further analysis of the CBI revealed that the perceived financial burden showed moderate positive correlations with the time-dependence burden (rho = 0.561, *p* < 0.001), developmental burden (rho = 0.529, *p* < 0.003), and physical burden (rho = 0.581, *p* < 0.001). However, no significant correlation was found between social (rho = 0.290, *p* = 0.075) and emotional (rho = 0.027, *p* = 0.448) burden (Table 4).

**Table 4:** Correlation of perceived financial burden, disability, QoL and caregiver burden*.

Scale/variable	Perceived financial burden		Disability	
	Rho (*r*)	*p*-value	Rho (*r*)	*p*-value
CHU-9D	−0.658	0.007	−0.643	0.009
Health utility score	−0.566	0.027	−0.629	0.014
Level of health state	0.362	0.124	0.732	0.003
OSSS-3	0.106	0.315	−0.131	0.276
Level of social support	0.293	0.088	−0.129	0.279
PHQ-9	0.624	0.009	0.425	0.065
Level of depression	0.461	0.048	0.184	0.265
CBI	0.526	0.003	0.476	0.007
Level of caregiver burden	0.503	0.004	0.420	0.016
Time-dependence burden	0.561	0.001	0.633	0.000
Developmental burden	0.529	0.003	0.343	0.043
Physical burden	0.581	0.001	0.409	0.019
Social burden	0.290	0.076	0.155	0.225
Emotional burden	0.027	0.446	0.337	0.046
PedsQL	−0.555	0.024	−0.806	0.000
Physical functioning	−0.321	0.143	−0.769	0.001
Emotional functioning	−0.368	0.108	−0.477	0.050
Social functioning	−0.504	0.039	−0.813	0.000
School functioning	−0.619	0.038	−0.694	0.019
Psychosocial functioning	−0.555	0.024	−0.827	0.000
Diagnostic delay	−0.399	0.020	−0.005	0.489
Disability	0.339	0.042	–	–

Correlation for variables in the study was determined using Spearman’s correlation.

*Sample sizes for correlation analyses varied depending on the scales completed by each participant. *N* = 13 for CHU-9D, *N* = 12 for EQ-5D-3L, *N* = 23 for OSSS-3, *N* = 14 for PHQ-9, *N* = 26 for CBI, *N* = 13 for PedsQL. r, correlation coefficient; QoL, Quality of life; CHU-9D, Child health utility-9 dimension; OSSS-3, Oslo social support scale-3; PHQ-9, Patient health questionnaire-9; CBI, Caregiver burden inventory; PedsQL, Pediatric quality of life inventory.

The ANOVA analysis revealed significant differences in the perceived financial burden for CHU-9D (*F* = 8.035, *p* = 0.006), PedsQL total score (*F* = 4.588, *p* = 0.033), and its subdomains such as social functioning (*F* = 4.423, *p* = 0.036) and psychosocial functioning (*F* = 4.701, *p* = 0.031). Moreover, the CBI total score (*F* = 7.430, *p* = 0.001), level of caregiver burden (*F* = 5.970, *p* = 0.004), time-dependence burden (*F* = 11.319, *p* = 0.000), developmental burden (*F* = 6.761, *p* = 0.002), and physical burden (*F* = 4.980, *p* = 0.009) all displayed significant differences in perceived financial burden.

### 3.7 Correlation of disability and QoL scales

In the study, out of 27 participants, 14 (51.9%) had multiple disabilities, 7 (25.9%) reported having one disability, and 6 (22.2%) had no disabilities (Figure 4E). The findings revealed that the presence of a disability had a moderate negative correlation with the CHU-9D scale (rho = −0.643, *p* < 0.009). Similarly, there was a strong negative correlation between the PedsQL total score (rho = −0.806, *p* < 0.000) and its subdomains, including physical functioning (rho = −0.769, *p* < 0.001), emotional functioning (rho = −0.477, *p* < 0.050), social functioning (rho = −0.813, *p* < 0.000), school functioning (rho = −0.694, *p* < 0.019), and psychosocial functioning (rho = −0.827, *p* < 0.000). The health utility score also demonstrated a moderate negative correlation (rho = −0.629, *p* < 0.014). Furthermore, the study identified a moderately positive correlation between the CBI total score (rho = 0.476, *p* < 0.014) and the level of caregiver burden (rho = 0.420, *p* < 0.016). Additionally, various aspects of caregiver burden, such as time-dependence burden (rho = 0.633, p < 0.000), developmental burden (rho = 0.343, *p* < 0.043), physical burden (rho = 0.409, *p* < 0.019), and emotional burden (rho = 0.337, *p* < 0.046), showed significantly positive correlations with the presence of disability (Table 4).

The ANOVA analysis revealed significant differences in the absence or presence of disability for several measures. Precisely, the CBI total score (*F* = 4.909, *p* = 0.017), level of caregiver burden (*F* = 3.452, *p* = 0.049), and time-dependence burden (*F* = 7.433, *p* = 0.003) all showed substantial differences. Similarly, the PedsQL total score (*F* = 14.931, *p* = 0.001) and its subdomains – physical functioning (*F* = 18.481, *p* = 0.000), social functioning (*F* = 11.344, *p* = 0.003), and psychosocial functioning (*F* = 9.380, *p* = 0.005) – also demonstrated meaningful distinctions. However, the CHU-9D score did not reach statistical significance (*F* = 3.862, *p* = 0.057).

## 4 Discussion

This study presents a thorough analysis of the clinical, disease burden, and quality of life (QoL) dimensions related to mitochondrial encephalomyopathy (ME), emphasizing its substantial effect on participants and their caregivers. The findings not only corroborate existing literature but also expand upon it, offering fresh insights into the intricate challenges posed by this rare disorder on participants’ quality of life and caregiver burden.

The study cohort was predominantly composed of children, who accounted for 88.9% of participants, with a mean age of 10.14 years. This suggests an early onset of ME (31, 32). Notably, common subtypes such as MELAS and Leigh syndrome were identified in 22.2% of the cases, underscoring the necessity for specialized pediatric care (7, 31, 33). While ME typically presents in childhood, participant ages varied from 1 month to 36 years, encompassing adult-onset conditions like Leber’s Hereditary Optic Neuropathy (LHON) (34). The diagnostic delay of approximately 1.9 years underscores a notable gap in the early recognition and intervention of mitochondrial diseases, despite significant advancements in genetic and clinical diagnostics (35).

Participants exhibited a range of symptoms, with the most prevalent being motor disability (25.9%) and delayed motor development (22.2%). The management of these symptoms had a negative impact on the QoL in both physical and psychosocial domains, consistent with previous research (36, 37). The most common mutations in this cohort included the m.3243A > G mutation in MELAS and mutations in both mitochondrial and nuclear genes in participants with Leigh syndrome. The m.3243A > G mutation was associated with an increased incidence of stroke-like episodes, corroborating past reports (4, 38). Additionally, participants with MELAS mutations displayed a variable range of symptoms at onset, including fatigue, muscle weakness, and cognitive impairment, frequently noted among MELAS participants (2). In contrast, participants diagnosed with Leigh syndrome, often resulting from mutations in both mitochondrial and nuclear genes, demonstrated a more pronounced early-onset neurological decline, characterized by severe cognitive impairment and significant motor disabilities. This aligns with clinical expectations that nuclear mutations typically lead to more severe and early-onset manifestations (7, 39). Additionally, approximately 25.9% of participants reported a family history of ME, suggesting a hereditary component, although this figure may not fully account for sporadic mitochondrial mutations.

Quality of life (QoL) was significantly impaired in all participants with ME across various scales, particularly in physical and emotional functioning domains. The mean scores of QoL assessment scales in our cohort were lower than the established standard norm, reflecting a considerable burden of disease (40, 41). Strong negative correlations were observed between QoL scores and perceived disability (rho = −0.643, *p* < 0.009), underscoring how functional limitations adversely affect quality of life. The presence of disability correlated with significantly lower scores on both the CHU-9D and PedsQL assessments, suggesting that genetic factors associated with disability contribute to diminished QoL outcomes. More than half of the participants (51.9%) reported experiencing multiple disabilities, which were linked to lower QoL and increased caregiver burden, consistent with findings from similar studies (42, 43). Disability among participants with ME was frequently related to reduced mobility, cognitive decline, and fatigue, all of which severely hindered their ability to perform daily activities. Additionally, 59.3% of participants reported discrimination related to their or their family members’ condition, while 78.3% indicated a lack of social support, highlighting the social isolation that individuals with rare diseases often endure. Social isolation is a well-known social determinant of health, particularly for individuals with chronic conditions and rare diseases. In the case of ME, the complex and progressive nature of the disease often leads to increased caregiver burden, which in turn isolates both participants and their families. The lack of social support exacerbates the emotional and psychological toll of the disease, as families struggle to access both practical and emotional assistance (44–46).

The financial burden associated with ME was a significant finding in this study, with 44.4% of participants and their families reporting severe financial stress. This burden is intensified by the high costs of treatment, including rehabilitation and long-term care, which are major contributors to their financial strain. A substantial majority of participants (88.9%) reported incurring debt related to medical expenses, with 33.3% owing between 30,000 and 60,000 CNY and 25.9% facing debt between 100,000 and 200,000 CNY. These financial challenges are consistent with other studies that show how rare diseases place considerable economic pressure on families (47).

Moreover, 59.3% of participants required full-time care, limiting caregivers’ ability to maintain employment and attend to their health needs. This finding aligns with existing research that indicates rare diseases often result in significant economic burdens, especially in healthcare systems with insufficient insurance coverage (47, 48). Additionally, the financial strain was found to have a negative correlation with QoL scores, particularly in the CHU-9D, PedsQL, and CBI domains, further affecting the well-being of participants and caregivers. The economic burden was strongly correlated with perceived disability (rho = 0.339, *p* < 0.042) and QoL scores (rho = −0.555, *p* < 0.024). This suggests that more severe disease leads to more significant financial and emotional strain. Nearly 58% of caregivers reported experiencing moderate to severe burdens. Caregivers reported higher scores in the time-dependence (rho = 0.633, *p* < 0.000), developmental burden (rho = 0.343, *p* < 0.043), and emotional burden (rho = 0.337, *p* < 0.046) domains. These results are consistent with previous studies indicating that caregivers of individuals with rare and severe neurological conditions experience significant emotional and physical strain (36, 37).

The need for rehabilitation services was evident, with 44.4% of participants requesting them. Nevertheless, high costs (33.3%) and a lack of local options (14.8%) posed significant barriers to accessing rehabilitation services. The demands of full-time caregiving further strained caregivers, leading to absenteeism and financial hardships. In our study, 57.7% of caregivers reported experiencing moderate to severe burdens, highlighting the physical, emotional, and time-related challenges that come with caregiving for individuals with ME (49, 50). These findings emphasize the urgent need for improved access to rehabilitation services and enhanced financial support for affected families.

Our findings underscore several actionable recommendations for clinical practice and policy development. First, the early identification of neuromuscular symptoms, especially motor retardation and seizures, should prompt clinicians to suspect mitochondrial dysfunction and refer participants for genetic testing in a timely manner. Second, the financial burden associated with ME calls for the establishment of enhanced insurance frameworks and financial assistance programs specifically designed for rare diseases. Third, developing comprehensive care models that incorporate genetic counseling, rehabilitation, mental health services, and social support is essential. These models should be informed by interdisciplinary collaborations and a patient-centered approach to adequately meet the diverse needs of people with ME and their families.

While this study offers valuable insights into the burden of ME on participants and their families, several limitations must be acknowledged. The small sample size of 27 participants limits the statistical power and generalizability of our findings, particularly due to the heterogeneity of mitochondrial encephalomyopathy subtypes, which encompass diverse clinical presentations and genetic mutations. Larger multicenter studies are needed to enhance the robustness and external validity of the results. Secondly, using self-reported measures to assess QoL and caregiver burden could introduce subjectivity and bias, as patient and caregiver perceptions may vary based on personal experiences, psychological states, and cultural factors. This could lead to overestimation or underestimation of certain aspects, such as the financial burden or the severity of disability. Moreover, using assessment tools validated for specific age ranges and depending on proxy responses for very young children and those with cognitive impairments may introduce biases that affect the accuracy of subjective experiences. Future research should aim to develop and utilize age- and developmentally appropriate instruments for young children and individuals with severe cognitive impairments to better assess their quality of life. Nevertheless, our study highlights the significant disease burden and poor quality of life faced by participants with ME and their caregivers.

## 5 Conclusion

In conclusion, enhancing the quality of life and long-term outcomes for individuals affected by mitochondrial encephalomyopathy requires a holistic approach that incorporates medical, psychological, and social support. By promoting early diagnosis, improving access to care, and implementing comprehensive policy measures, we can significantly enrich the lives of those impacted by this challenging condition. Further research into the genetic foundations, innovative therapies, and psychosocial support strategies will be crucial in advancing the care and assistance of individuals with ME, ultimately nurturing a better future for participants and their families.

## Data Availability

The original contributions presented in the study are included in the article/supplementary material, further inquiries can be directed to the corresponding authors.
